# HOTAIR regulates SIRT3-mediated cardiomyocyte survival after myocardial ischemia/reperfusion by interacting with FUS

**DOI:** 10.1186/s12872-023-03203-0

**Published:** 2023-03-30

**Authors:** Jixuan Liu, Mingzhuang Sun, Jinda Wang, Zhijun Sun, Gang Wang

**Affiliations:** 1 Department of Cardiovascular, Beijing Friendship Hospital, Capital Medical University, No.95, Yongan Road, Beijing, 100050 China; 2 Department of Cardiovascular, Aerospace Central Hospital, Beijing, 100853 China; 3 Department of Cardiology, The Sixth Medical Centre of PLA General Hospital, Beijing, 100853 China

**Keywords:** lncRNA HOTAIR, SIRT3, FUS, Myocardial ischemia/reperfusion, Cardiomyocyte survival

## Abstract

**Background:**

Myocardial ischemia/reperfusion (I/R) contributes to serious myocardial injury and even death. Therefore, prevention and mitigation of myocardial I/R is particularly important. LncRNA HOTAIR has been reported to be implicated in myocardial I/R progression. However, the detailed molecular mechanism of HOTAIR in cardiomyocyte was explored in myocardial I/R.

**Methods:**

Firstly, cell model of myocardial I/R was established through hypoxia/reoxygenation (H/R). Apoptosis and cell cycle were evaluated utilizing flow cytometry. The corresponding test kits were conducted to monitor the levels of LDH, Caspase3 and Caspase9. The gene expression and protein levels were detected by qPCR and western blot, respectively. RNA pull-down and RIP were performed to verify the interaction between FUS and lncRNA HOTAIR.

**Results:**

In AC16 cardiomyocytes treated with H/R, lncRNA HOTAIR and SIRT3 expression were obviously decreased. Overexpression of HOTAIR or SIRT3 could ameliorate H/R-induced cardiomyocyte injury by promoting cell viability, lowering LDH levels, and suppressing cell apoptosis. Further, lncRNA HOTAIR upregulated the expression of SIRT3 via interacting with FUS, thereby promoting the survival of H/R-injured cardiomyocytes.

**Conclusion:**

LncRNA HOTAIR can improve myocardial I/R by affecting cardiomyocyte survival through regulation of SIRT3 by binding to the RNA binding protein FUS.

**Supplementary Information:**

The online version contains supplementary material available at 10.1186/s12872-023-03203-0.

## Highlights

1. Overexpression of HOTAIR or SIRT3 promotes H/R-induced cardiomyocyte survival.

2. LncRNA HOTAIR regulates the expression of SIRT3 by binding to FUS.

3. HOTAIR/FUS/SIRT3 axis facilitates cardiomyocyte survival under H/R treatment.

**Supplementary Information:**

The online version contains supplementary material available at 10.1186/s12872-023-03203-0.

## Background

Myocardial infarction (MI) is the term used for the event of a heart attack, which occurs when plaque builds up in the lining of the arteries, resulting in reduced blood flow to the heart and damage to the heart muscle due to lack of oxygen [1]. It is mainly due to coronary artery occlusion and insufficient blood supply, leading to myocardial ischemic death and long-term ischemic necrosis of myocardial cells, and is the main cause of morbidity and death worldwide [2].The effective treatment for MI is reperfusion therapy, which can promote blood recovery in the ischemic myocardium. However, this treatment can cause ischemia/reperfusion (I/R) damage, which can lead to secondary and complex heart damage [3]. Accordingly, developing approaches to increase the cardiomyocyte survival is vital to attenuate myocardial I/R injury.

Long non-coding RNAs (lncRNAs) are a type of non-coding RNA with more than 200 nucleotides, which are involved in numerous processes of human physiology and pathology, such as the regulation of the cardiovascular system [4, 5]. Studies have shown that a large number of lncRNAs have a close regulatory relationship with the development of myocardial I/R [6, 7]. For example, study showed that lncRNA SNHG1 expression was reduced and overexpressing lncRNA SNHG1 improved the injury by cardiac I/R via mediating HIF-1α/VEGF pathway, indicating that lncRNA SNHG1 play a pivotal role in myocardial I/R injury [8]. LncRNA Hox transcript antisense intergenic RNA (HOTAIR), as an eye-catching lncRNA, has been extensively studied in a variety of diseases, including cancer, ischemic stroke, epilepsy and myocardial I/R [9, 10]. It was reported that HOTAIR expression was downregulated in the serum of acute MI patients and mouse model [11]. Overexpression of HOTAIR protected from MI and hypoxia-induced cardiomyocyte apoptosis, indicating HOTAIR was negatively associated with disease risk of incident MI [12, 13]. However, others suggested that HOTAIR aggravated myocardial I/R injury and promoted inflammation after acute MI [14, 15]. Therefore, the functions and molecular mechanisms of lncRNA HOTAIR deserve to be further explored in MI. In addition, there is evidence that lncRNAs can regulate downstream targets by interacting with RNA-binding proteins and participate in MI progression [16]. Through bioinformatics prediction, it was found that lncRNA HOTAIR has a potential binding relationship with fused in sarcoma (FUS), and FUS has been reported to be involved in MI-related myocardial injury [16]. SIRT3, an NAD-dependent protein deacetylase, mainly present in mitochondria and plays a pivotal role in regulating cell apoptosis [17]. SIRT3 was downregulated in post-infarct myocardial injury and its overexpression can alleviate myocardial fibrosis, maintain myocardial function, inhibit inflammatory response, and reduce myocardial cell death [18, 19]. However, whether HOTAIR interacts with FUS to regulate SIRT3 expression requires further research. Taken together, we hypothesize that HOTAIR regulates SIRT3-mediated cardiomyocyte survival after myocardial I/R by interacting with FUS.

## Materials and methods

### Cell culture and treatment

Human cardiomyocyte cell line AC16 was obtained from American Type Culture Collection (ATCC, Manassas, VA, USA) and cultured in Dulbecco's modified Eagle's medium (DMEM; Sigma-Aldrich, St. Louis, MO, USA) supplemented with 10% fetal bovine serum, 1% penicillin and 1% streptomycin under condition of a humidified chamber with 5% CO_2_ and at 37 ℃.

Hypoxia/reoxygenation (H/R) AC16 cardiomyocyte model was constructed by the following protocols: cells were cultured under the condition of 37 °C, 5% CO_2_, 10% H_2_ and 85% N_2_ for 4, 8, 12, 16 and 20 h, followed reoxygenation treatment for 3 h with 5% concentration of CO_2_ and 95% concentration of O_2_. Cells cultured under normal condition (5% CO_2_ and 95% air) were used as controls [20].

### Cell transfection

For overexpression of HOTAIR (oe- HOTAIR), SIRT3 (oe-SIRT3) and FUS (oe-FUS), cells were transfected with pcDNA3.1-HOTAIR/FUS/SIRT3 plasmids (Invitrogen, Carlsbad, CA, USA). Empty vector (pcDNA3.1, oe-NC) was used as the negative control. All cell transfections were performed by using Lipofectamine™ 3000 (Invitrogen) according to the manufacturer’s instruction. In brief, cells (1 × 10^5^) were inoculated into each well in a six-well plate and cultured until cell confuence reached 60–70%. Then, target plasmids (4 µg) and 10 µL of Lipofectamine 3000 were diluted with 250 µL of serum-free medium. Next, the mixture was added to the culture system. After 6 h in culture, the medium was replaced with complete culture medium.

After a further 48 h, cells were collected to detect the transfection efciency for subsequent experiments.

### Cell viability assay

Cell Counting Kit-8 (CCK-8; Dojindo, Kumamoto, Japan) assay was employed to test the viability of AC16 cardiomyocytes. The cells were planted into a 96 plate at a density of 1000 cells/well. After 24 h incubation, each well was added 10 μL CCK-8 solution and incubated for 4 h at 37 °C. Then, a microplate reader (Thermo Fisher Scientific, Waltham, MA, USA) was employed to measure the absorbance at 450 nm after the CCK-8 reaction.

### Western blot

The total protein was extracted from AC16 cardiomyocytes using RIPA buffer (Beyotime, Shanghai, China) and the protein concentration was tested using the Bicinchoninic acid (BCA) protein quantification kit (Beyotime). Sodium dodecyl sulfate‐polyacrylamide gel electrophoresis (SDS‐PAGE) was employed to separate the proteins (30 μg/sample), and then the proteins in SDS-PAGE were transferred onto the polyvinylidene difluoride (PVDF) membrane (Millipore, Billerica, MA, USA). After blocking with 5% bovine serum albumin (BSA) for 1 h, membrane was cut according to the molecular weight of the target proteins and incubated with primary antibodies that were purchased from Abcam (Cambridge, MA, USA), including Cyclin D1 (1:200, ab16663), p21 (1:2000, ab109520), Bcl-2 (1:2000, ab182858), Bax (1:5000, ab32503), FUS (1:1000, ab124923), SIRT3 (1:1000, ab217319) and GAPDH (1:2500, ab9485) overnight at 4 °C. Subsequently, the membrane was incubated with the HRP-conjugated secondary antibody (1:10,000, ab6721) for 1 h. Enhanced chemiluminescence (ECL) reagent (Beyotime) were applied to visualize protein bands. The gray value analysis was evaluated by using Image J software.

### Quantitative real‐time PCR (qPCR)

TRIzol reagent (Invitrogen) was performed to extract RNA from AC16 cardiomyocytes. Prime Script Reverse Transcription Reagent Kit (TaKaRa, Tokyo, Japan) was conducted to synthesized cDNA, SYBR Premix Ex Taq II Kit (TaKaRa) was used in qPCR. Relative RNA expression was calculated by using 2^−ΔΔt^ formula. GAPDH was regarded as reference gene. The primers used in this study were listed as follows:

- HOTAIR forward: 5′-GCCTTTCCCTGCTACTTGTG-3′,
- HOTAIR reverse: 5′-AGAGCTTCCAAAGGCTAGGG-3′;
- SIRT3 forward: 5′-CGTTGTGAAGCCCGACATTG-3′,
- SIRT3 reverse: 5′-CACCAAGTCCCGGTTGATGA-3′;
- Cyclin D1 forward: 5′-AGCTGTGCATCTACACCGAC-3′,
- Cyclin D1 reverse: 5′-GAAATCGTGCGGGGTCATTG-3′;
- p21 forward: 5′-AGGTGGACCTGGAGACTCTCAG-3′,
- p21 reverse: 5′-TCCTCTTGGAGAAGATCAGCCG-3′;
- Bcl-2 forward: 5′-GATGACTGAGTACCTGAACC-3′,
- Bcl-2 reverse: 5′-AGTTCCACAAAGGCATCC-3′;
- Bax forward: 5′-CTGACGGCAACTTCAACTGGG-3′,
- Bax reverse: 5′-CAACCACCCTGGTCTTGGATC-3′;
- FUS forward: 5′-CAGACAGGGAAACTGGCAAGCT-3′,
- FUS reverse: 5′-GGCGAGTAGCAAATGAGACCTTG3′;
- GAPDH forward: 5′-GTCTCCTCTGACTTCAACAGCG-3′,
- GAPDH reverse: 5′-ACCACCCTGTTGCTGTAGCCAA-3′.

### Flow cytometry analyses

Annexin V-FITC/ propidium iodide (PI) staining kit (Invitrogen) was used to evaluate apoptosis. AC16 cardiomyocytes were harvested with trypsin digestion solution, washed twice with phosphate-buffered saline (PBS) and incubated with Annexin V-FITC (5 µL) and PI solution (10 µL) for 15 min. Subsequently, the cell apoptosis rate was quantitatively assessed by FACScan flow cytometer (Beckman Coulter, USA).

For cell cycle detection, AC16 cardiomyocytes at the logarithmic phase were collected, centrifuged and resuspended at 2 × 10^6^ cells/mL. Then the cells were incubated in 70% ethanol for 30 min at 4 °C. After staining with a PI solution containing 0.1 mg/mL RNase A, 50 µg/mL PI, and 0.2% Triton X-100, cell cycle was determined by using flow cytometry and analysed using FlowJo software.

### The measurement of LDH levels

The levels of LDH in AC16 cardiomyocytes with corresponding disposal were evaluated by the LDH assay kit ((Nanjing Jiancheng, China) according to the manufacturer instructions. A microplate reader was employed to monitor the optical density at 450 nm.

### Caspase 3 and caspase 9 activity assays

The activities of caspase3 and caspase9 in AC16 cardiomyocytes were determined by Caspase3 Activity Assay Kit (C1116, Beyotime) and Caspase9 Activity Assay Kit (C1158, Beyotime) as the manufacturer's instructions. The reaction mixtures were incubated at 37 °C for 2 h followed by detection of the caspase3 and caspase9 activities at 405 nm in a microtiter plate reader.

### RNA immunoprecipitation (RIP) assay

The association of HOTAIR with FUS was detected by the EZMagna RIP Kit (Millipore). In short, 5 × 10^6^ cells were treated with RIP lysis buffer for 30 min at 4 °C. Then, RIP buffer (500 μL) containing magnetic beads (50 μL) conjugated to antibodies (5 μL) against anti-FUS antibody (ab234880, Abcam), SNRNP70 (positive control; ab83306, Abcam) or immunoglobulin (Ig) G (negative control; Abcam) for 24 h at 4 °C. The HOTAIR in immunoprecipitation complexes was detected by qPCR.

### RNA pull-down assay

HOTAIR was labeled with biotin (50 nM) using the Biotin RNA Labeling Mix (Roche, Basel, Switzerland), which were incubated with RNase-free TURBO DNase I (Invitrogen) and Sephadex G-50 Quick Spin Columns (Sigma-Aldrich). Then, cells (1.5 × 10^7^) were lysed in IP buffer (200 μL; 20 mM Tris–HCl, pH 8.0, 200 mM NaCl, 1 mM EDTA, 1 mM EGTA, 0.5% Triton X-100, 0.4 U/μL RNasin), followed by 30-min incubation with biotinylated HOTAIR. Then, the supernatant cell lysate was collected by centrifugation, each lysate was added with an equal amount of streptavidin magnetic beads (Thermo Fisher Scientific) and incubated for 30 min at room temperature. After magnetic bead separation in a magnetic stand and sample elution with elution buffer at 37 °C on a shaker for 30 min, the FUS protein in the HOTAIR-protein complexes was analyzed by western blot.

### mRNA stability assay

As for detection of mRNA stability of SIRT3, treated cells were administrated with actinomycin D (5 μg/mL) for 0, 3 and 6 h. After that, RNA was extracted and reverse transcribed into cDNA for qPCR measurement.

### Statistical analyses

All data collected from at least three experiments were presented in the form of mean ± standard deviation (SD). All statistical analyses were calculated using GraphPad Prism (version 8.0). A comparison between the two groups were statistically detected using Student's t-test and more than two groups was evaluated by one-way ANOVA followed by Tukey's post hoc test. When* p* < 0.05, there were significant differences between the data.

## Results

### The expression of HOTAIR and SIRT3 were declined in H/R-induced cardiomyocytes

**Fig. 1:**
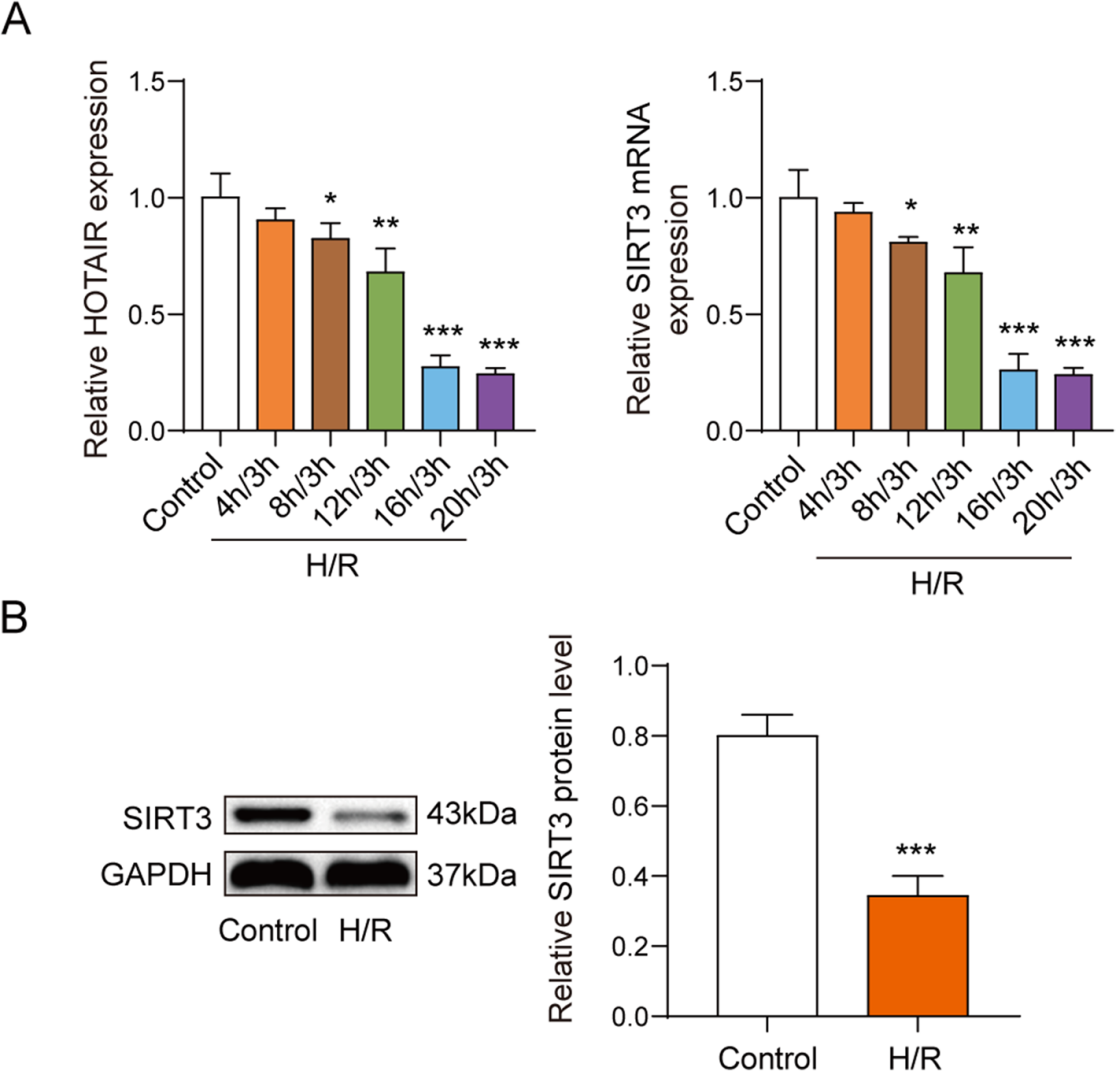
LncRNA HOTAIR and SIRT3 expression were downregulated in H/R-induced cardiomyocytes. AC16 cardiomyocytes were cultured under the condition of hypoxia for 0, 8, 12, 16 and 20 h followed by reoxygenation for 3 h to constructed cell model of MI. **A** qPCR results showed that lncRNA HOTAIR and SIRT3 expression were decreased in a time-dependent mannerunder the condition of hypoxia. **B** Western blot results showed that the expression level of SIRT3 protein decreased with the increase of hypoxia time. * *p* < 0.05, ** *p* < 0.01, *** *p* < 0.001

For understanding the levels of key genes in MI, AC16 cardiomyocytes were subjected to the hypoxia condition for 4, 8, 12, 16 and 20 h at 37℃ respectively followed by reoxygenation for 3 h. Subsequently, we found the expression levels of lncRNA HOTAIR and SIRT3 were decreased in a time-dependent manner with maximum effect at 16 h (Fig. 1A). Therefore, hypoxia for 16 h and reoxygenation for 3 h was selected for following experiments. Further, the protein level of SIRT3 in H/R-induced cardiomyocytes was significantly lower than that in normal cardiomyocytes (Fig. 1B).

### Overexpression of HOTAIR or SIRT3 promoted H/R-induced cardiomyocyte survival

**Fig. 2:**
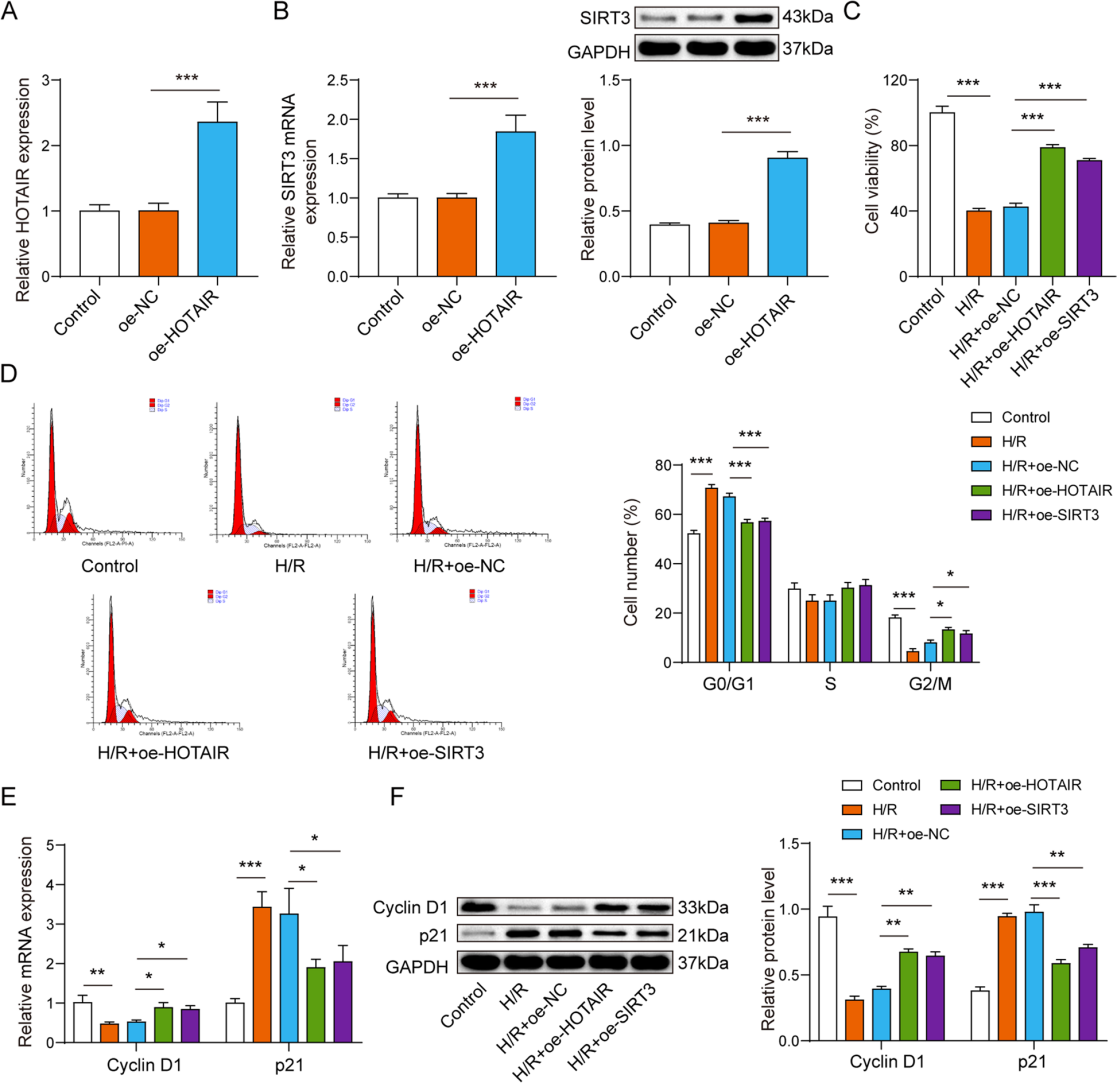
Overexpression of lncRNA HOTAIR or SIRT3 promoted H/R-induced cardiomyocyte survival. **A**-**B** qPCR indicated that oe-HOTAIR and oe-SIRT3 were transfected successfully. **C** CCK-8 assay illustrated that viability of AC16 cardiomyocytes was reduced under induction of H/R, but overexpression of HOTAIR or SIRT3 reversed this effect. **D** The flow cytometry results showed that overexpression of HOTAIR or SIRT3 significantly inhibited H/R-induced G0/G1 phase arrest and activated the G2/M cell cycle transition. **E** The qPCR assay indicated that Overexpression of HOTAIR or SIRT3 significantly upregulated the expression of Cyclin D1 and downregulated the expression of p21 in H/R-induced cardiomyocytes. **F** Western blot asssay results showed the same trend. * *p* < 0.05, ** *p* < 0.01, *** *p* < 0.001

Aiming to reveal the effects of HOTAIR and SIRT3 on cardiomyocyte survival, AC16 cardiomyocytes which underwent with H/R were transfected with oe-NC, oe-HOTAIR and oe-SIRT3. Firstly, the results show successful overexpression of HOTAIR and SIRT3 (Fig. 2A-2B). Regarding biological functions, viability of AC16 cardiomyocytes was reduced under induction of H/R, but overexpression of HOTAIR or SIRT3 reversed this effect (Fig. 2C). Concurrently, the cell cycle status was detected by flow cytometry, and the results showed that overexpression of HOTAIR or SIRT3 significantly inhibited H/R-induced G0/G1 phase arrest and activated the G2/M cell cycle transition (Fig. 2D). Overexpression of HOTAIR or SIRT3 significantly upregulated the expression of Cyclin D1 and downregulated the expression of p21 in H/R-induced cardiomyocytes (Fig. 2E-2F), overexpression of HOTAIR or SIRT3 significantly upregulated the expression of Cyclin D1 and downregulated the expression of p21 in H/R-induced cardiomyocytes. Taken together, lncRNA HOTAIR and SIRT3 are protective for I/R injury in MI by promoting cell viability and activating the G2/M cell cycle transition.

### Overexpressing HOTAIR or SIRT3 inhibited H/R-induced cell apoptosis

**Fig. 3:**
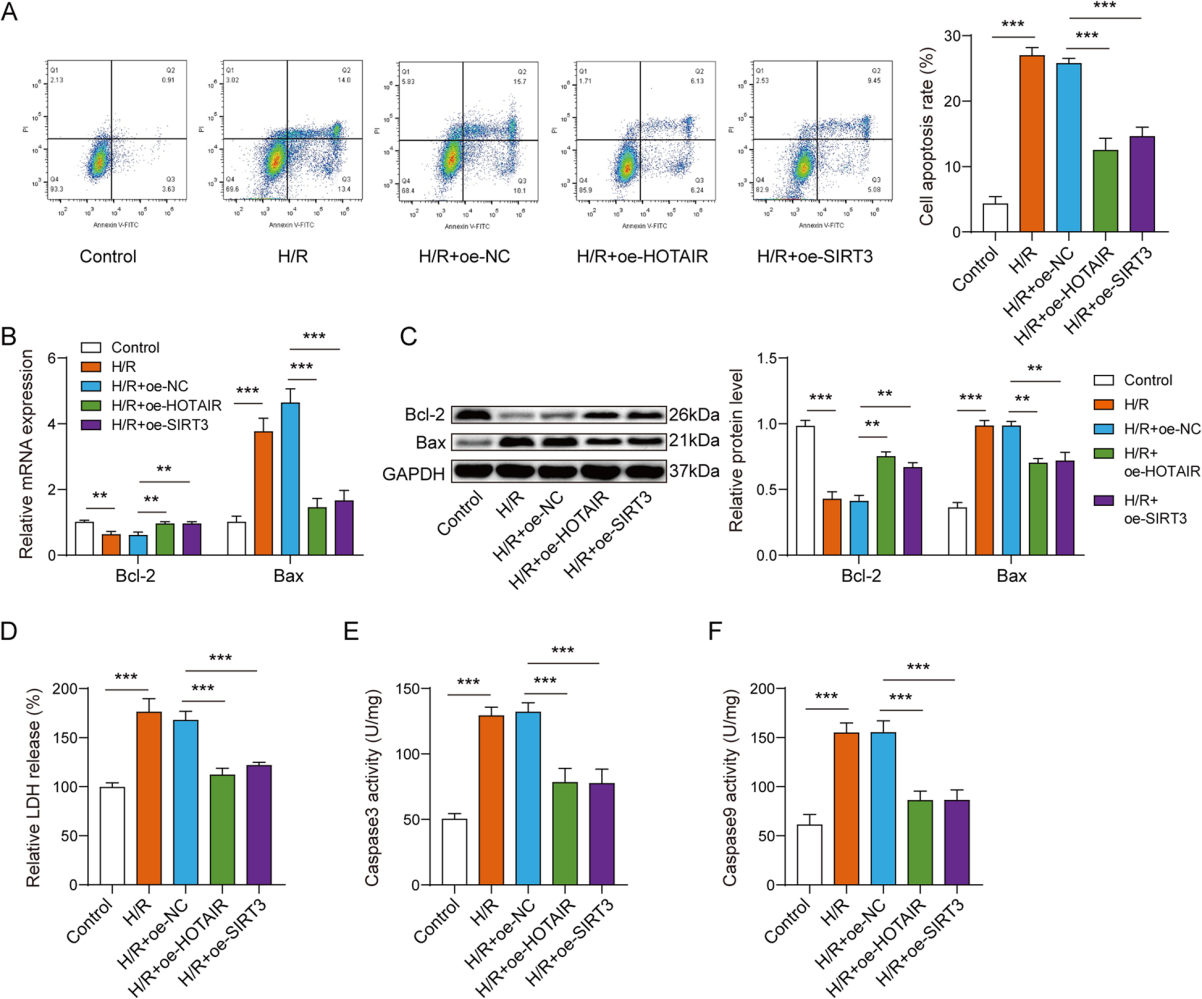
Overexpressing lncRNA HOTAIR or SIRT3 inhibited H/R-induced cell apoptosis. **A** The flow cytometry results showed the apoptosis rate of cardiomyocytes in H/R model was increased, and overexpression of HOTAIR or SIRT3 significantly inhibited H/R-induced cardiomyocyte apoptosis. **B** The qPCR (**C**) and western blot assay showed that the upregulation of Bax and descent of Bcl-2 in H/R-induced AC16 cardiomyocytes were notably attenuated by overexpressing HOTAIR or SIRT3. The level of LDH (**D**) and (**E**–**F**) caspase3 and caspase9 showed overexpression of HOTAIR or SIRT3 reduced the increased activities of LDH, caspase3 and caspase9 in H/R-induced cardiomyocyte model ** *p* < 0.01, *** *p* < 0.001

Next, the role of lncRNA HOTAIR and SIRT3 in cell apoptosis were assessed. Flow cytometry results showed that the apoptosis rate of cardiomyocytes in H/R model was increased compared with normal cardiomyocytes, and overexpression of HOTAIR or SIRT3 significantly inhibited H/R-induced cardiomyocyte apoptosis (Fig. 3A). In addition, the upregulation of Bax and descent of Bcl-2 in H/R-induced AC16 cardiomyocytes were notably attenuated by overexpressing HOTAIR or SIRT3 (Fig. 3B-C). Meanwhile, overexpression of HOTAIR or SIRT3 reduced the increased activities of LDH, caspase3 and caspase9 in H/R-induced cardiomyocyte model (Fig. 3D-F). In total, lncRNA HOTAIR or SIRT3 reduced H/R-induced cell apoptosis, lowered LDH levels and also reduced caspase3 and caspase9 activities..

### LncRNA HOTAIR regulated the expression of SIRT3 by binding to FUS

**Fig. 4:**
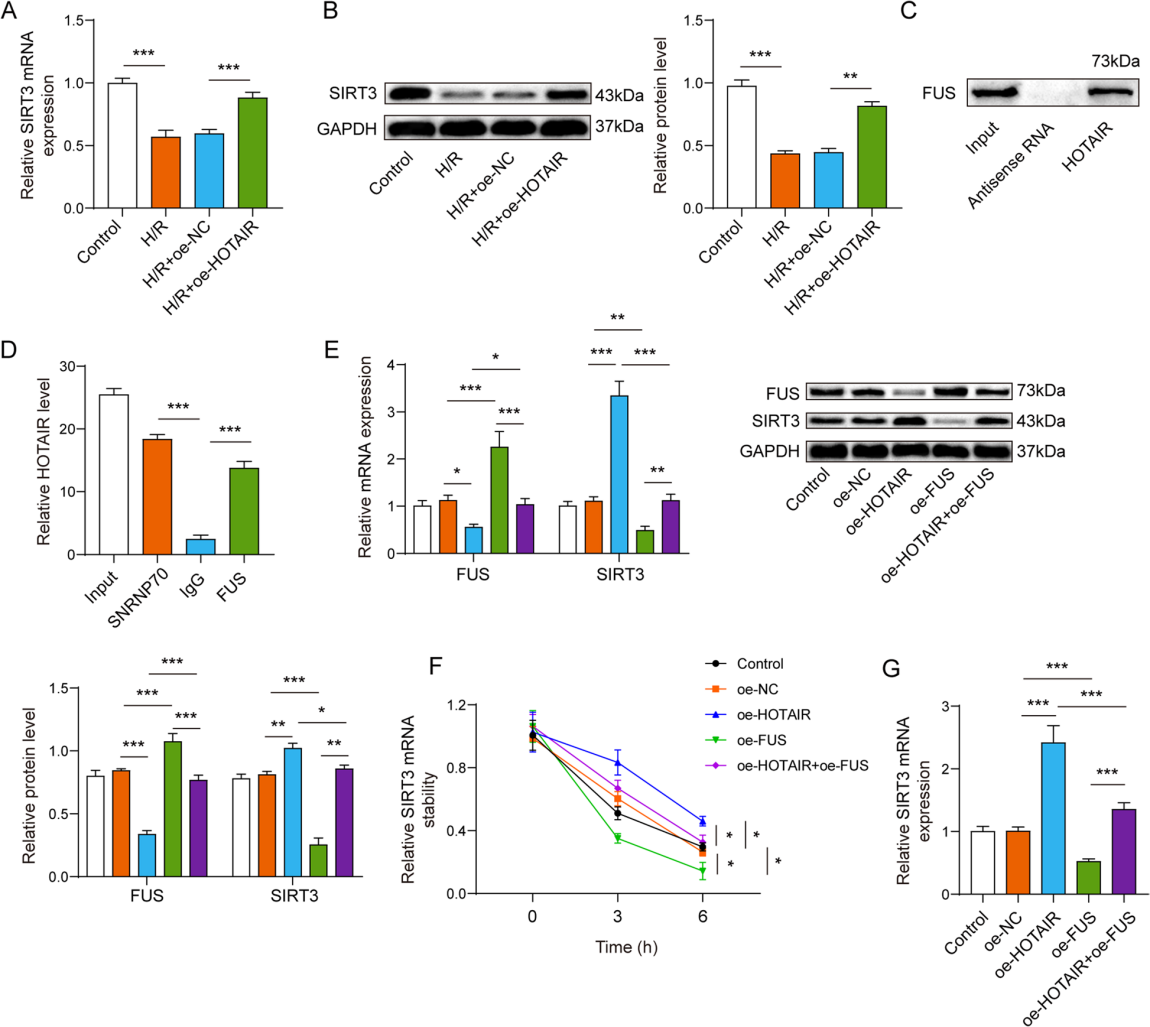
LncRNA HOTAIR regulated the expression of SIRT3 by binding to FUS. The qPCR (**A**) and western blot (**B**) showed overexpression of HOTAIR resulted in higher expression of SIRT3 in H/R-treated cells. RNA pull-down (**C**) and RIP (**D**) validated the binding relationship between HOTAIR and FUS. **E** Western blot showed that oe-HOTAIR significantly up-regulated the expression of SIRT3 and downregulated the expression of FUS, and oe-FUS could reverse the promotion of SIRT3 by oe-HOTAIR. **F** The mRNA decay of SIRT3 was accelerated by oe-FUS transfection, while was inhibited by oe-HOTAIR in the presence of actinomycin D. **G** The mRNA expression of SIRT3 was upregulated by oe-HOTAIR, while downregulated by oe-FUS. *p < 0.05, ** *p* < 0.01, *** *p* < 0.001

Then, we want to test whether and how HOTAIR regulates SIRT3 expression. Firstly, we observed H/R-decreased SIRT3 expression and overexpression of HOTAIR resulted in higher expression of SIRT3 (Fig. 4A-B). Besides, to unveil the relation between HOTAIR and FUS, RNA pull-down assay verified the binding of HOTAIR to FUS. (Fig. 4C). Conformably, the results from RIP displayed that HOTAIR was apparently enriched by FUS immunoprecipitation (Fig. 4D). Additionally, we also noticed that oe-HOTAIR significantly up-regulated the expression of SIRT3 and down-regulated the expression of FUS, and oe-FUS could reverse the promotion of SIRT3 by oe-HOTAIR (Fig. 4E). Then, the mRNA decay of SIRT3 was accelerated by oe-FUS transfection in the presence of actinomycin D, however, HOTAIR overexpression could promote stability of SIRT3 mRNA, which could reverse by oe-FUS (Fig. 4F). Consistently, oe-HOTAIR increased SIRT3 mRNA expression, while oe-FUS decreased SIRT3 mRNA expression and partly reversed the effects of oe-HOTAIR on SIRT3 mRNA expression (Fig. 4G). To sum up, lncRNA HOTAIR may regulate SIRT3-mediated cytoprotection by interacting with FUS.

### LncRNA HOTAIR regulated H/R-induced cardiomyocyte survival via the FUS/SIRT3 axis

**Fig. 5:**
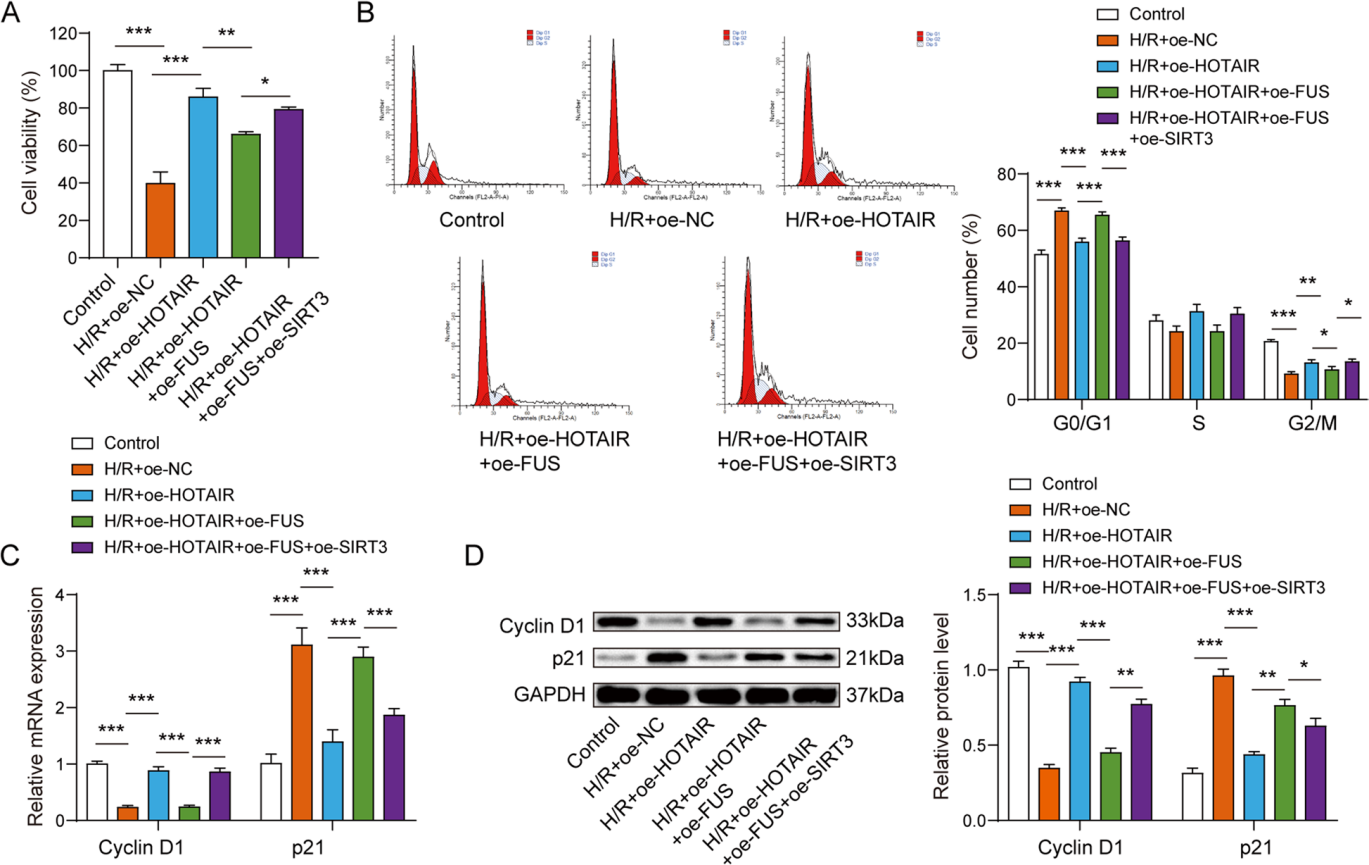
LncRNA HOTAIR regulated H/R-induced cardiomyocyte survival via the FUS/SIRT3 axis. **A** CCK-8 assay showed oe-FUS can reverse the promoting effect of lncRNA HOTAIR on the cell viability of H/R cardiomyocyte model, while overexpression of SIRT3 suppressed the effect of overexpressing FUS. **B** Flow cytometry indicated that overexpression of FUS partially reversed the effect of HOTAIR on cell G0/G1 arrest, while overexpression of SIRT3 inhibited the effect of FUS. The qPCR (**C**) and western blot assay (**D**) showed that lncRNA HOTAIR overexpression promoted Cyclin D1 and decreased p21 expression. And overexpression of FUS can partially reverse the effect of overexpression of HOTAIR, while overexpression of SIRT3 can inhibit the effect of oe-FUS. *p < 0.05, ** *p* < 0.01, *** *p* < 0.001

To clarify the effect of FUS/SIRT3 axis on lncRNA HOTAIR-mediated cell survival in MI, oe-HOTAIR, oe-FUS, oe-SIRT3 alone or oe-HOTAIR together with oe-FUS or oe-HOTAIR together with oe-FUS and oe-SIRT3 was transfected into AC16 cardiomyocytes following H/R treatment. When lncRNA HOTAIR was overexpressed in AC16 cardiomyocytes following H/R treatment, cell viability was significantly increased compared with the H/R model. oe-FUS can reverse the promoting effect of lncRNA HOTAIR on the cell viability of H/R cardiomyocyte model, while overexpression of SIRT3 suppressed the effect of overexpressing FUS (Fig. 5A). Then, flow cytometry was used to detect cell cycle, the results indicated that overexpression of FUS partially reversed the effect of HOTAIR on cell G0/G1 arrest, while overexpression of SIRT3 inhibited the effect of FUS (Fig. 5B). Moreover, we observed that lncRNA HOTAIR overexpression promoted Cyclin D1 expression and decreased p21 expression in H/R-induced AC16 cardiomyocytes. Nevertheless, overexpression of FUS can partially reverse the effect of overexpression of HOTAIR on Cyclin D1 and p21, while overexpression of SIRT3 can inhibit the effect of oe-FUS (Fig. 5C-5D). Additionally, the effects of HOTAIR on cell viability, Cyclin D1 and p21 expressions were partly reversed by SIRT3 silence (Fig. 7A-7B). In conclusion, lncRNA HOTAIR promoted cell survival by regulating SIRT3 via binding to FUS in H/R-induced AC16 cardiomyocytes.

### LncRNA HOTAIR regulated H/R-induced cell apoptosis via FUS/SIRT3 axis

**Fig. 6:**
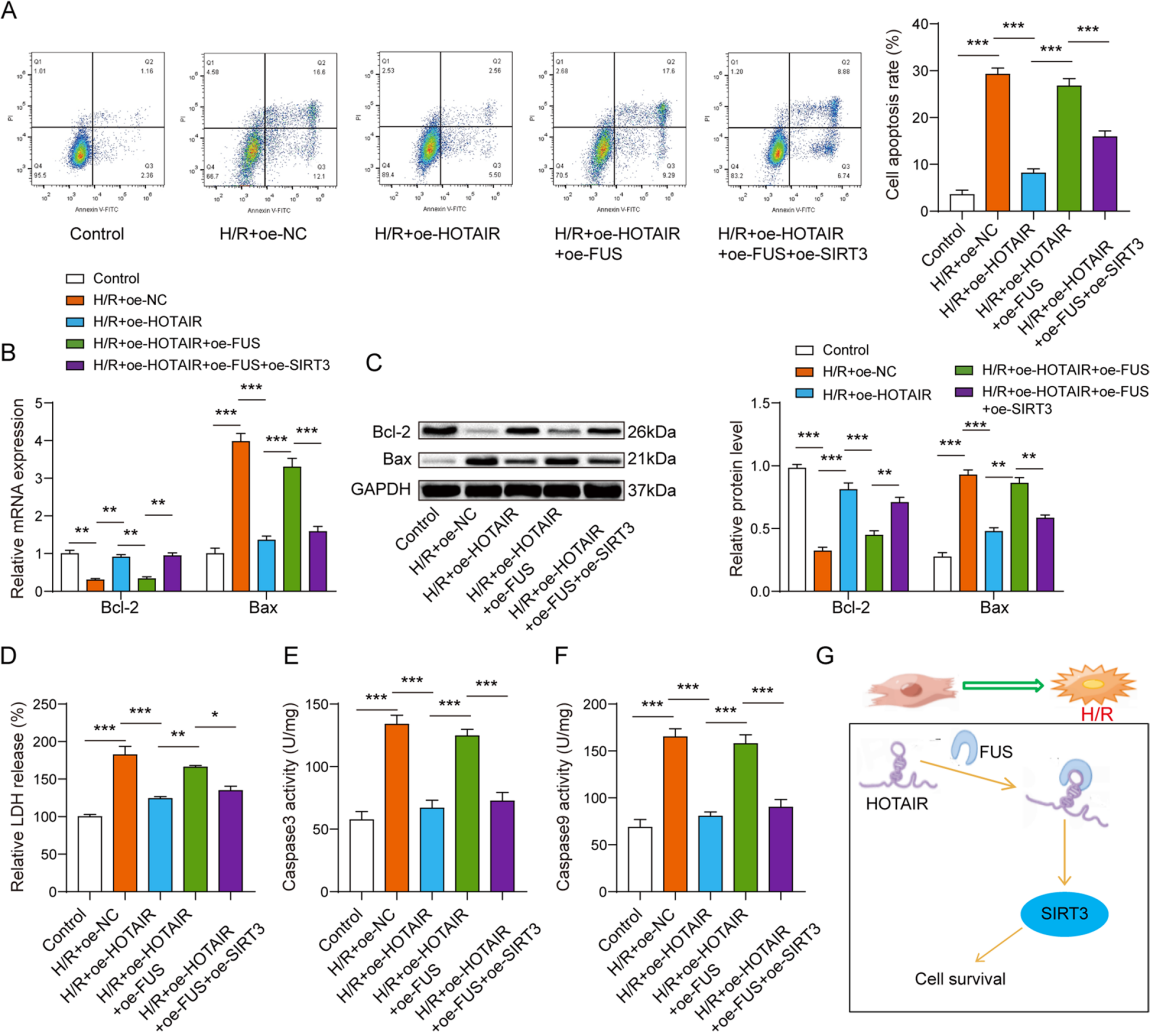
LncRNA HOTAIR regulated H/R-induced cell apoptosis via FUS/SIRT3 axis. **A** Cell apoptosis was reduced by overexpressing HOTAIR, and overexpression of FUS could reverse the suppressing effect of oe-HOTAIR on apoptosis. The qPCR (**B**) and western blot assay (**C**) showed that oe-FUS could reverse the Bcl-2 and Bax expression levels in oe-HOTAIR treated H/R cardiomyocyte model. The levels of LDH (**D**), caspase3 and caspase9 (**E**–**F**) was dramatically decreased by oe-HOTAIR in H/R-induced AC16 cardiomyocytes, however, combined with oe-FUS transfection, these phenomena were counteracted. **G** Schematic diagram of the mechanism of HOTAIR regulating cardiomyocyte survival. *p < 0.05, ** *p* < 0.01, *** *p* < 0.001

**Fig. 7:**
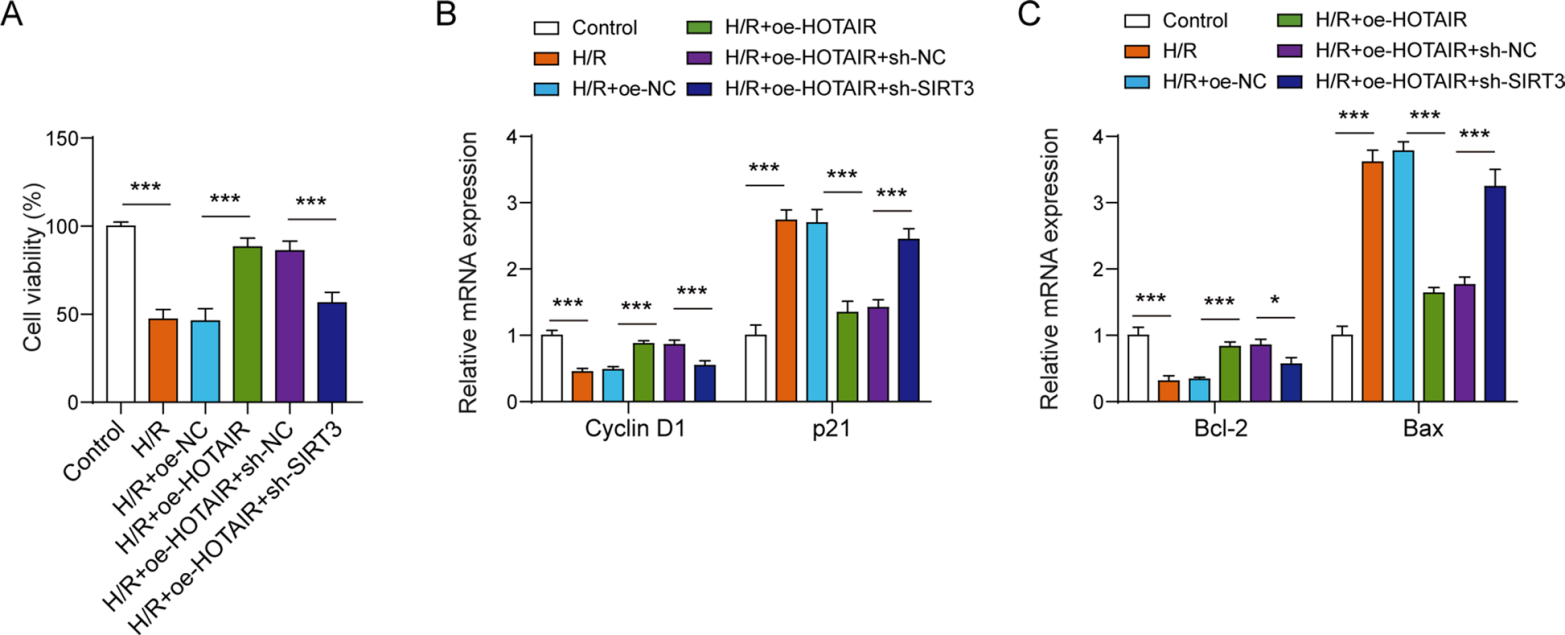
Effects of SIRT3 inhibition on the roles of HOTAIR in cell viability and cycle/apoptosis molecule expressions. **A** Cell viability was increased by overexpressing HOTAIR, and inhibition of SIRT3 could suppress the effect of HOTAIR on cell viability. **B** The qPCR assay showed that knockdown of SIRT3 partially reversed the effect of HOTAIR on Cyclin D1 and p21 expressions. **C** The results of qPCR showed that silencing of SIRT3 partly reversed the effect of HOTAIR on Bcl-2 and Bax expressions. *p < 0.05, *** *p* < 0.001

Cell apoptosis was reduced by overexpressing HOTAIR in H/R-induced AC16 cardiomyocytes, and overexpression of FUS could reverse the suppressing effect of oe-HOTAIR on apoptosis in H/R cardiomyocyte model. However, combined with transfection of oe-SIRT3, this phenomenon was partially cancelled out (Fig. 6A). When lncRNA HOTAIR was over-expressed in AC16 cardiomyocytes following H/R treatment, Bcl-2 expression was obvious higher while Bax expression was notably lower than that in AC16 cardiomyocytes following H/R treatment. oe-FUS could reverse the Bcl-2 and Bax expression levels in oe-HOTAIR treated H/R cardiomyocyte model. But this effect of oe-FUS was reversed by oe-SIRT3 transfection (Fig. 6B-6C). Moreover, the levels of LDH, caspase3 and caspase9 was dramatically decreased by oe-HOTAIR in H/R-induced AC16 cardiomyocytes, however, combined with oe-FUS transfection, these phenomena were counteracted. Moreover, the effect of oe-FUS was reversed by oe-SIRT3 transfection (Fig. 6D-F). Further, inhibition of SIRT3 could partially reversed the effects of HOTAIR on Bcl-2 and Bax expressions (Fig. 7C). Collectively, lncRNA HOTAIR regulated the expression of SIRT3 by interaction with FUS, thus inducing myocardial cell apoptosis inhibition and promotion of cell survival (Fig. 6G).

## Discussion

MI may result in myocardial cell apoptosis and necrosis, as well as cardiac arrest and other serious consequences, which greatly increases the lethality of this disease [21]. Myocardial I/R in MI are closely related to cardiomyocyte death. Here, we investigated the specific molecular mechanism of lncRNA HOTAIR in myocardial I/R injury, that is, lncRNA HOTAIR ameliorated H/R-induced AC16 cardiomyocyte injury by regulating SIRT3 expression via interacting with the RNA-binding protein FUS.

Growing evidence points to the importance of lncRNAs in myocardial I/R [22, 23]. Different lncRNAs have different effects on myocardial I/R by involving regulation of cell viability, apoptosis, inflammatory cytokines. Studies have shown lncRNA ANRIL against H/R injury in H9C2 cardiomyocytes [20], and lncRNA Malat1 regulated microvascular function after MI in mice via miR-26b-5p/Mfn1 axis-mediated mitochondrial dynamics [24]. In the current study, we confirmed the beneficial effects of lncRNA HOTAIR on H/R-induced AC16 cardiomyocytes by promoting cell viability, reducing the release of LDH and inhibiting apoptosis. A previous study reported that lncRNA HOTAIR protected MI rats by sponging miR-519d-3p [13]. In addition, the HOTAIR/miR-17-5p axis is involved in the protection of propofol-mediated myocardial I/R injury [25]. Although the function of lncRNA HOTAIR in MI has been investigated, the specific mechanism remains unclear.

In general, lncRNAs can interact with RNA-binding proteins and participate in the regulation of target gene expression [26]. It is well known that RNA-binding proteins are an important class of proteins in cells [27]. They are widely involved in RNA splicing, transport, sequence editing, intracellular localization and translation by recognizing special RNA binding domains and interacting with RNA. It can regulate the expression of target genes during multiple post-transcriptional regulatory processes [28]. A study has shown that circFndc3b interacted with RNA-binding protein FUS in sarcomas to decrease FUS level, thereby upregulating vascular endothelial growth factor (VEGFA) expression and activating VEGFA signaling [29]. FUS could interact with downstream mRNA to silence its expression [30]. In the present study, lncRNA HOTAIR bound to FUS and negatively regulated its expression. HOTAIR upregulated SIRT3, while FUS inhibited SIRT3 expression. Thus, HOAIR may competitively bind FUS to promote SIRT3 expression and affects the survival and death of cardiomyocytes. Furthermore, the regulatory mechanism of HOTAIR or FUS in SIRT3 deserves further exploration in future researches.

SIRT3 played an important role cardiac repair and remodeling after MI by targeting proteins associated with oxidative stress, I/R injury, mitochondrial metabolic homeostasis, and cell death [19]. Additionally, studies have shown that overexpression of SIRT3 can inhibit p21 and promote the expression of Cyclin D1 to alleviate cell senescence [31, 32], which were consistent with our results. p21 and Cyclin D1 are closely related to cell survival and proliferation. SIRT3 was reported to induce p53 deacetylation to downregulate p53 expression [33]; p53, a transcription factor, could bind to the p21 promoter to increase p21 transcription [34]. Besides, p53 can decrease Cyclin D1 transcription through downregulation of Bcl-3 and inducing increased association of the p52 NF-kappaB subunit with histone deacetylase 1 [35]. These findings indicated that SIRT3 may repress p21 expression and upregulate Cyclin D1 by modulating p53 acetylation level. In the present study, it was observed that overexpression of SIRT3 alleviated H/R-induced cardiomyocyte death, thereby counteracting the damaging effects of H/R on cardiomyocytes. However, the functional mechanisms of SIRT3 need to be further explored and verified.

In summary, we describe a novel molecular regulatory mechanism in myocardial I/R, whereby the lncRNA HOTAIR binds to FUS to regulate SIRT3 expression to suppress H/R-induced cardiomyocyte injury, enriching the theory of myocardial I/R and providing new targets for the treatment of myocardial I/R. However, there are still some limitations in this study. For example, we only provide experimental data at the cellular level, and neither animal experiments nor clinical samples are included in this study. Further studies at the in *vivo* level and downstream signaling pathways in myocardial I/R are needed to increase the impact of the research.

## Supplementary Information

**Additional file 1:** **Supplementary Figure 1.** Theoriginal blots of Fig. 1B were presented. **Supplementary Figure 2.** (A) The original blots of Fig. 2B were presented. (B) The original blots of Fig. 2F were presented. **Supplementary Figure 3.** The original blots of Fig. 3C were presented. **Supplementary Figure 4.** (A) The original blots of Fig. 4B were presented. (B) The original blots of Fig. 4C were presented. (C) The original blots of Fig. 4E were presented. **Supplementary Figure 5.** The original blots of Fig. 5D were presented. **Supplementary Figure 6.** The original blots of Fig. 6C were presented.

## Data Availability

The datasets used or analyzed during the current study are available from the corresponding author on reasonable request.
