# Supplementary material for: HOTAIR regulates SIRT3-mediated cardiomyocyte survival after myocardial ischemia/reperfusion by interacting with FUS
Source: BMC Cardiovasc Disord. 2023 Mar 30;23:171. doi: 10.1186/s12872-023-03203-0 (PMC10061961; doi:10.1186/s12872-023-03203-0)
Supplement: Supplementary file 1 — Additional file 1: Supplementary Figure 1. Theoriginal blots of Fig. 1B were presented. Supplementary Figure 2. (A) The original blots of Fig. 2B were presented. (B) The original blots of Fig. 2F were presented. Supplementary Figure 3. The original blots of Fig. 3C were presented. Supplementary Figure 4. (A) The original blots of Fig. 4B were presented. (B) The original blots of Fig. 4C were presented. (C) The original blots of Fig. 4E were presented. Supplementary Figure 5. The original blots of Fig. 5D were presented. Supplementary Figure 6. The original blots of Fig. 6C were presented. [file 12872_2023_3203_MOESM1_ESM.ppt]

## Slide 1
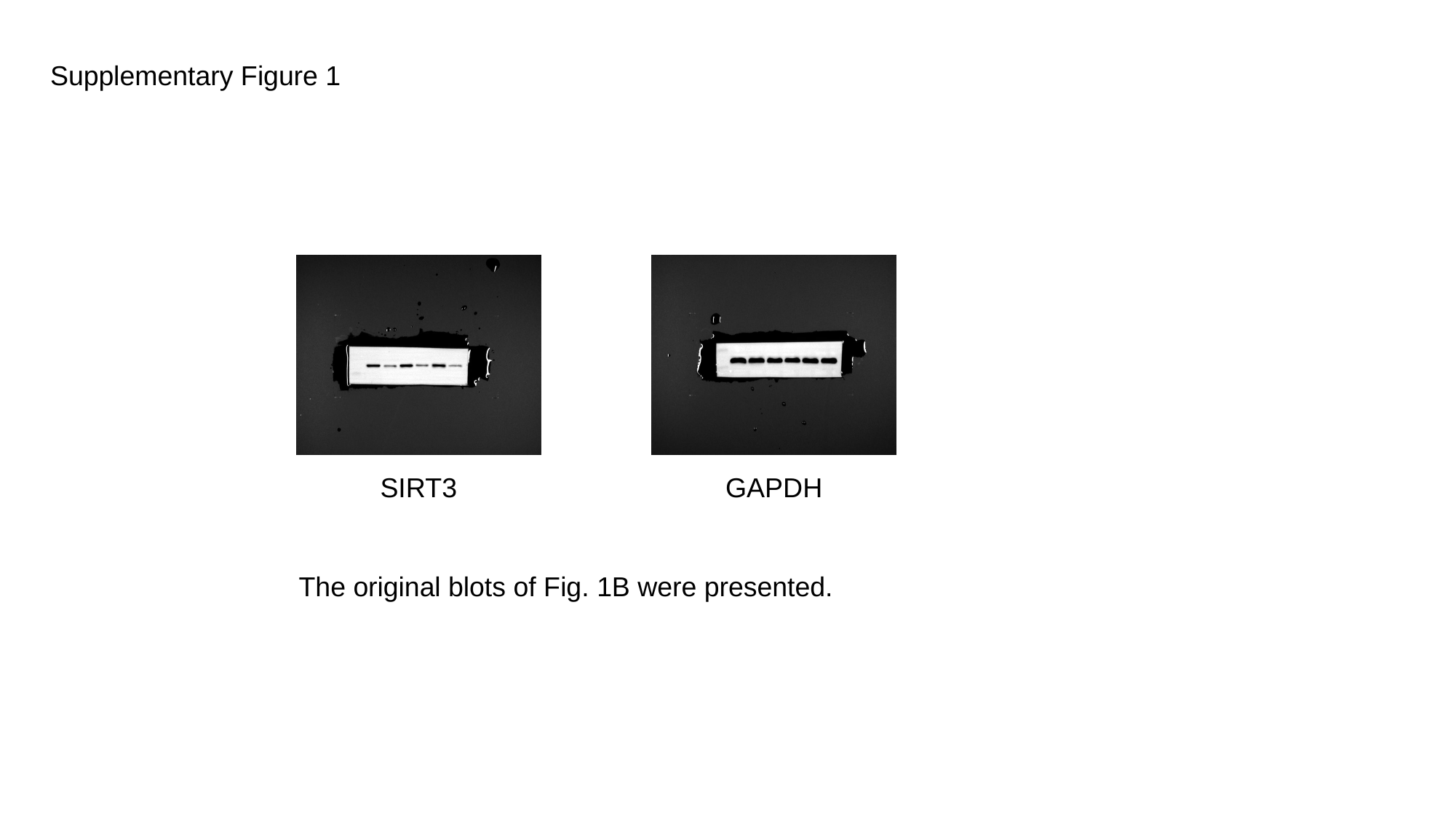

Supplementary Figure 1
SIRT3
GAPDH
The original blots of Fig. 1B were presented.

## Slide 2
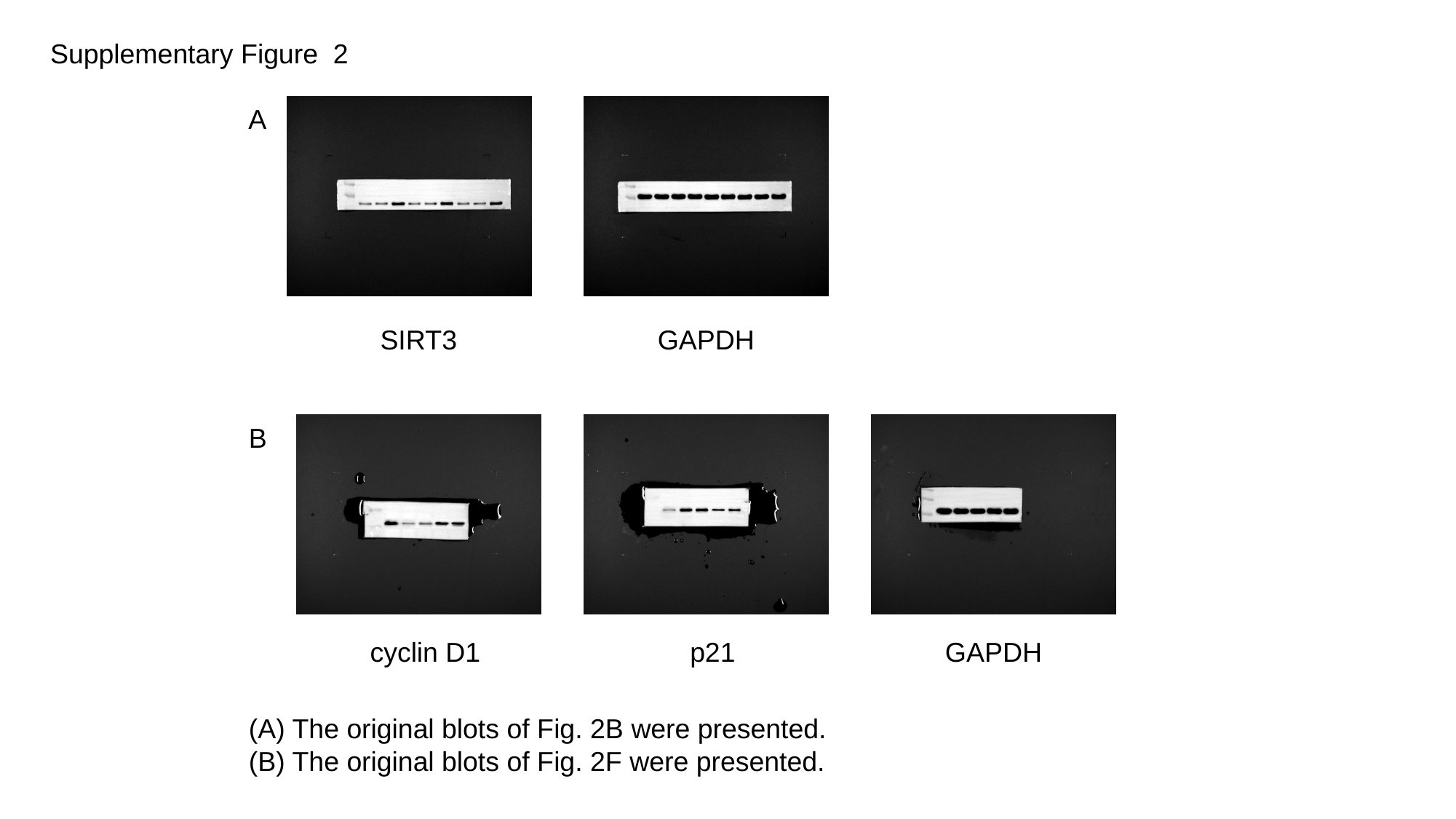

Supplementary Figure 2
A
SIRT3
GAPDH
B
cyclin D1
p21
GAPDH
(A) The original blots of Fig. 2B were presented. (B) The original blots of Fig. 2F were presented.

## Slide 3
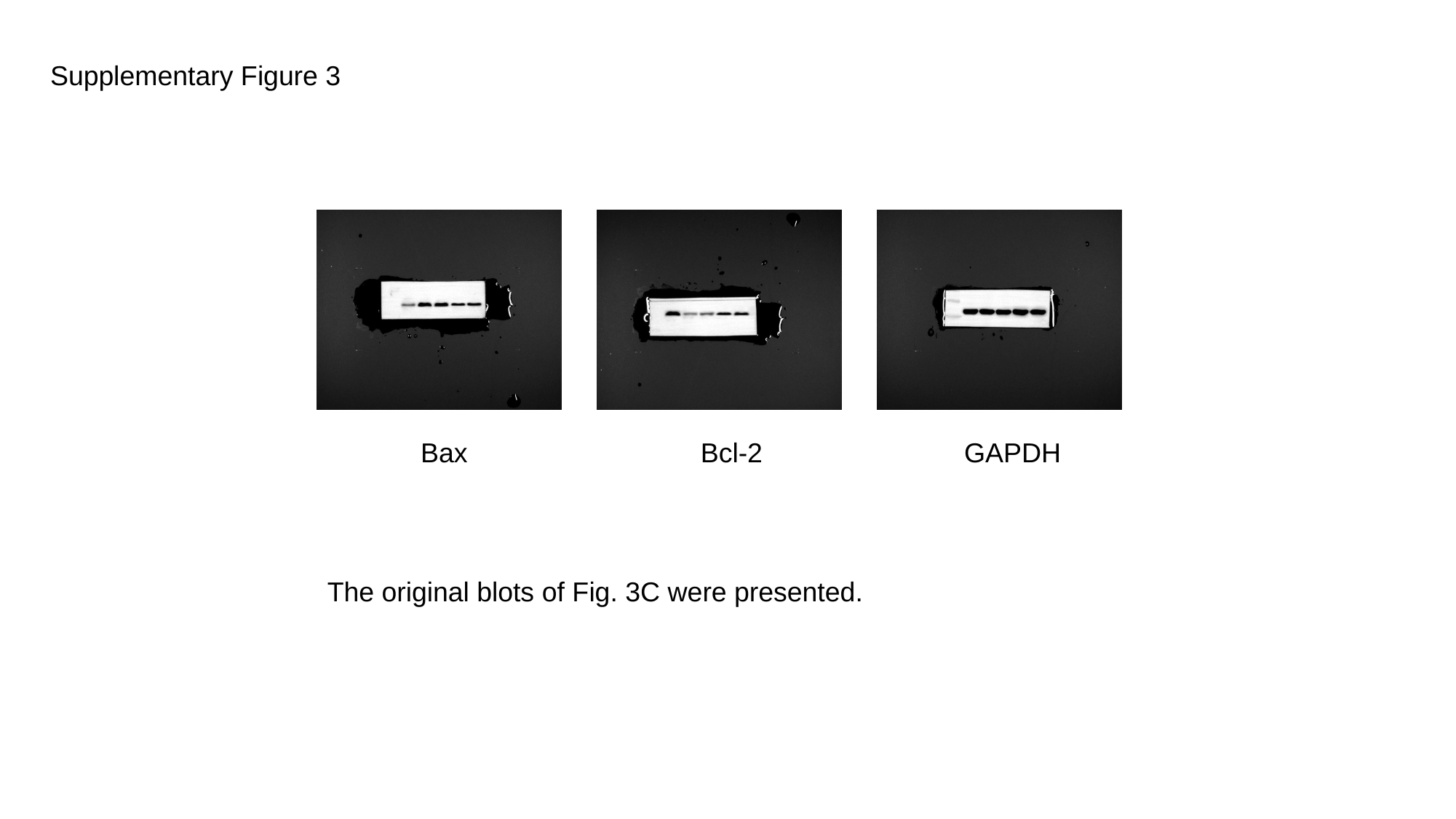

Supplementary Figure 3
Bax
Bcl-2
GAPDH
The original blots of Fig. 3C were presented.

## Slide 4
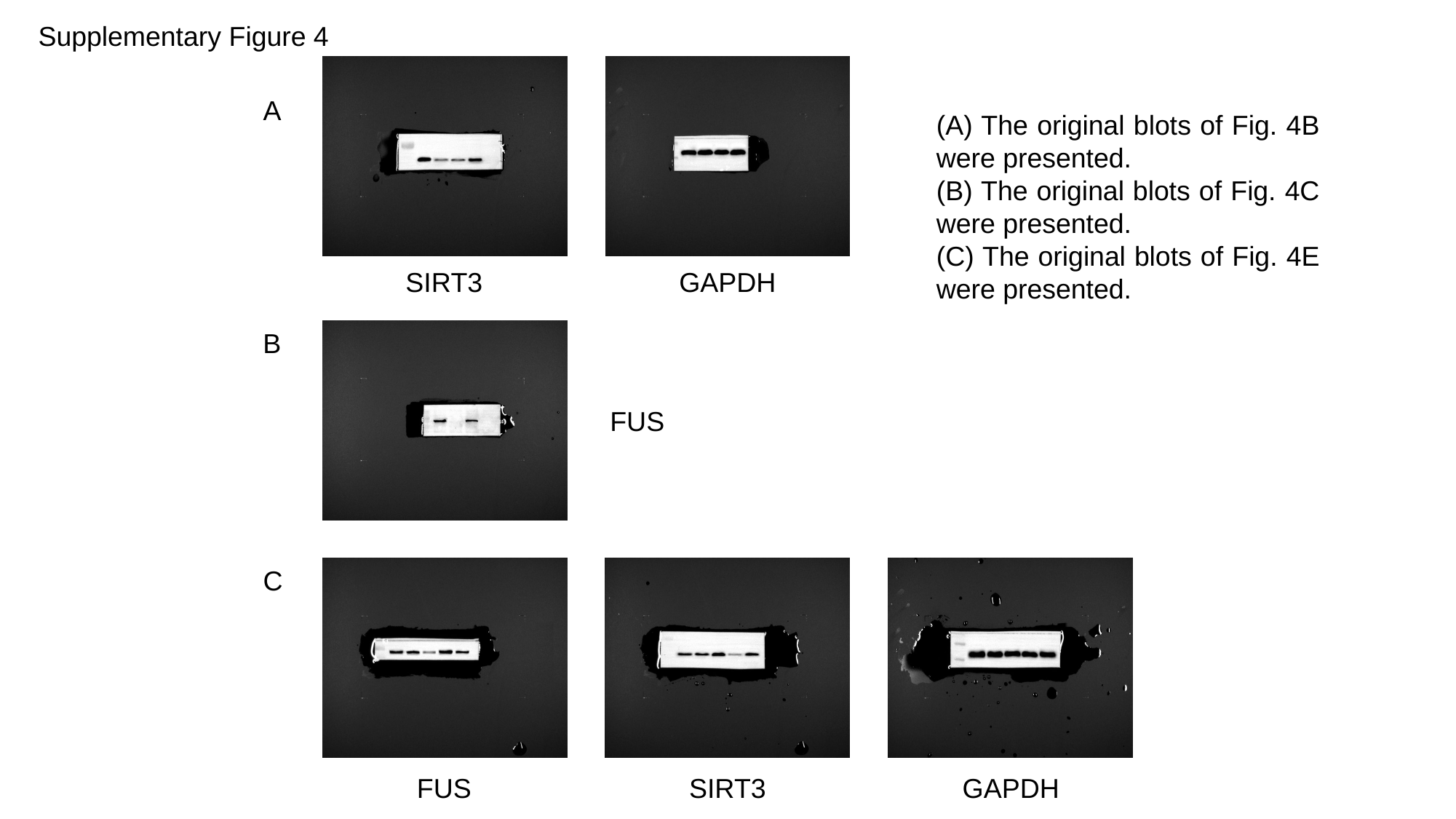

Supplementary Figure 4
A
(A) The original blots of Fig. 4B were presented.
(B) The original blots of Fig. 4C were presented.
(C) The original blots of Fig. 4E were presented.
SIRT3
GAPDH
B
FUS
C
FUS
SIRT3
GAPDH

## Slide 5
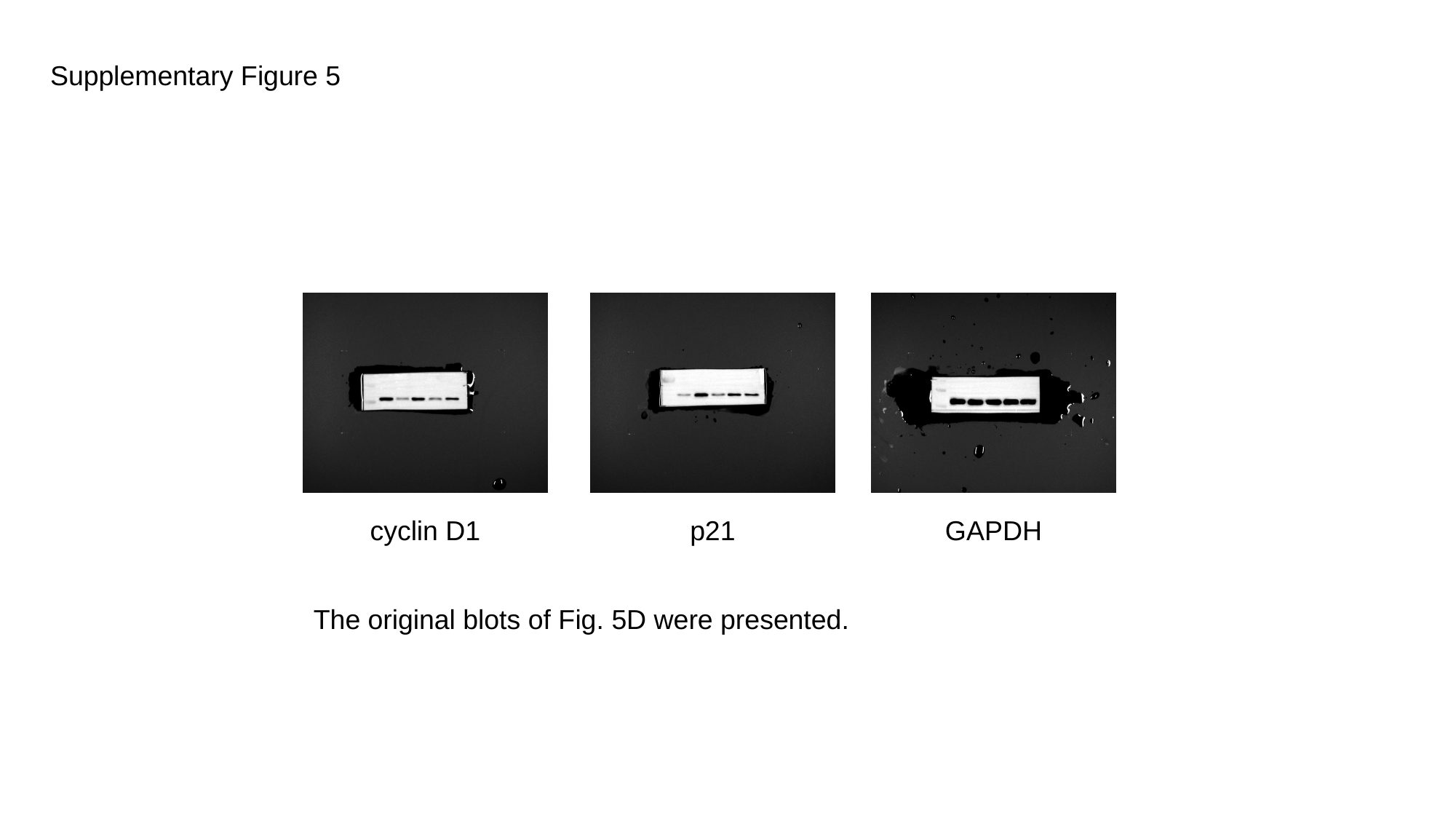

Supplementary Figure 5
cyclin D1
p21
GAPDH
The original blots of Fig. 5D were presented.

## Slide 6
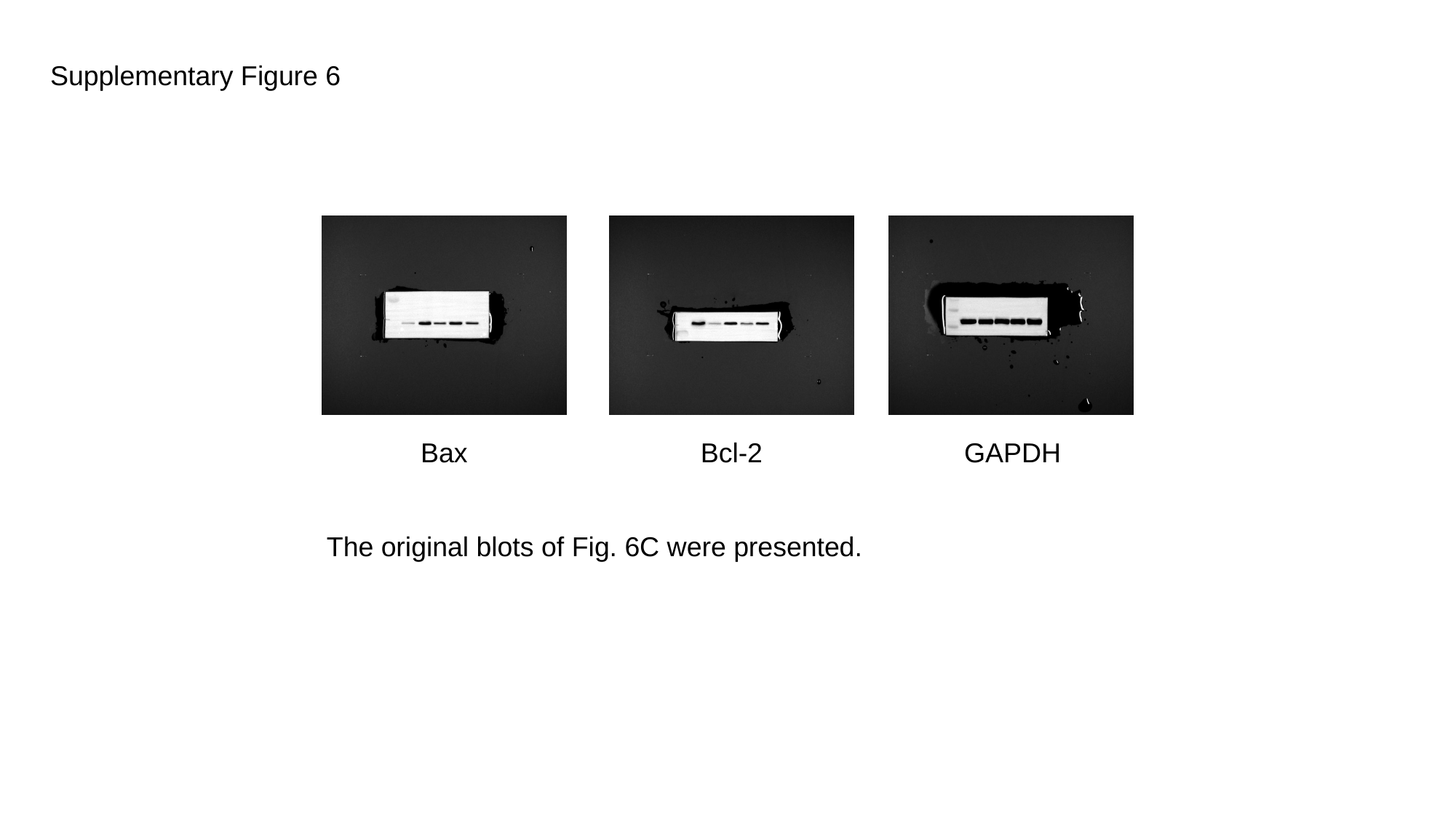

Supplementary Figure 6
Bax
Bcl-2
GAPDH
The original blots of Fig. 6C were presented.
